# Human memory T cell dynamics after aluminum-adjuvanted inactivated whole-virion SARS-CoV-2 vaccination

**DOI:** 10.1038/s41598-023-31347-8

**Published:** 2023-03-21

**Authors:** Ece Tavukcuoglu, Hamdullah Yanik, Mubaida Parveen, Sila Uluturk, Mine Durusu-Tanriover, Ahmet Cagkan Inkaya, Murat Akova, Serhat Unal, Gunes Esendagli

**Affiliations:** 1 Department of Basic Oncology, Hacettepe University Cancer Institute, 06100 Sihhiye, Ankara, Turkey; 2 Department of Infectious Diseases and Clinical Microbiology, Faculty of Medicine, Hacettepe University, Ankara, Turkey; 3 Department of Internal Medicine, Faculty of Medicine, Hacettepe University, Ankara, Turkey

**Keywords:** Vaccines, Inactivated vaccines

## Abstract

This study evaluates the functional capacity of CD4^+^ and CD8^+^ terminally-differentiated effector (T_EMRA_), central memory (T_CM_), and effector memory (T_EM_) cells obtained from the volunteers vaccinated with an aluminum-adjuvanted inactivated whole-virion SARS-CoV-2 vaccine (CoronaVac). The volunteers were followed for T cell immune responses following the termination of a randomized phase III clinical trial. Seven days and four months after the second dose of the vaccine, the memory T cell subsets were collected and stimulated by autologous monocyte-derived dendritic cells (mDCs) loaded with SARS-CoV-2 spike glycoprotein S1. Compared to the placebo group, memory T cells from the vaccinated individuals significantly proliferated in response to S1-loaded mDCs. CD4^+^ and CD8^+^ memory T cell proliferation was detected in 86% and 78% of the vaccinated individuals, respectively. More than 73% (after a short-term) and 62% (after an intermediate-term) of the vaccinated individuals harbored T_CM_ and/or T_EM_ cells that responded to S1-loaded mDCs by secreting IFN-γ. The expression of CD25, CD38, 4-1BB, PD-1, and CD107a indicated a modulation in the memory T cell subsets. Especially on day 120, PD-1 was upregulated on CD4^+^ T_EMRA_ and T_CM_, and on CD8^+^ T_EM_ and T_CM_ cells; accordingly, proliferation and IFN-γ secretion capacities tended to decline after 4 months. In conclusion, the combination of inactivated whole-virion particles with aluminum adjuvants possesses capacities to induce functional T cell responses.

## Introduction

Since the beginning of the coronavirus disease 2019 (COVID-19) pandemic, immunity against severe acute respiratory syndrome coronavirus 2 (SARS-CoV-2) has been widely investigated^1^. In severe cases, lymphopenia and excessive proinflammatory cytokines such as interleukin (IL)-1β, IL-6, and tumor necrosis factor (TNF)-α were observed in line with the activation of innate and adaptive immunity^2–4^. In contrast to many other respiratory viral infections, lymphopenia in COVID-19 can be selective to T cell lineage^5–7^. Only limited numbers of T cells infiltrate the lungs while cytotoxic T lymphocytes (CTLs) are significantly decreased in the upper respiratory tract^6^.

T cell-mediated immunity is pivotal for the elimination of viral infections. T cells orchestrate and regulate the immune responses, clear the infected cells, augment the presentation of viral antigens, and maintain the high-affinity antibody production by B lymphocytes^8^. Therefore, the protective immunity is provided not only through the B cells and the antibody-producing plasma cells but also through the long-lived memory T cells^9^. Not underscoring the essential role of antibody-mediated responses, the evidence from COVID-19 patients and/or from the individuals vaccinated for SARS-CoV-2 indicated the significance of T cell-mediated immunity^10^. Functionality of T cell memory responses secures the robustness of recall immunity against SARS-CoV-2 and may provide cross-reactivity for distinct antigenic variants of the virus^11^.

The receptor binding domain (RBD) of SARS-CoV-2 spike (S) glycoprotein is the main target for neutralization; hence, it became a common component in many COVID-19 vaccines^12,13^. The promise of mRNA vaccines, viral vector vaccines and subunit vaccines fueled the research on protective immunity and immunological memory^14,15^. On the other hand, disadvantages of the conventional vaccines, which generally consist of an inactivated pathogen, or a component of the pathogen admixed with adjuvants, have been discussed in COVID-19 pandemic^16,17^. Hypothetically, protective immunity induced by inactivated whole-virion vaccines may remain partial since the immune response will not exclusively focus on the antigen(s) responsible of viral entry^18,19^. In addition, the aluminum-adjuvanted vaccines have been unsatisfactory for establishing interferon (IFN)-γ-mediated immunity and T cell memory^20,21^.

Upon completion of a double-blind, randomized, placebo-controlled phase 3 vaccine trial in Turkey which demonstrated considerable efficiency against symptomatic COVID-19^22^, a group of volunteers were enrolled in this study. Here, we aimed to investigate the functional capacities of the memory and effector T cells induced by an aluminum-adjuvanted inactivated whole-virion SARS-CoV-2 vaccine, CoronaVac. The short-term (on day 7) and intermediate-term (on day 120) responses of cytotoxic and helper T cells against SARS-CoV-2 spike glycoprotein S1 were tested following the second dose of the vaccine.

## Materials and methods

### Participants and vaccination protocol

The participants from a randomized, case-driven phase 3 clinical trial (registered clinical trials No. NCT04582344), which was initiated on Sept 15, 2020, and terminated on Jan 13, 2021, were enrolled. A group of volunteers who participated in the phase 3 clinical trial was followed up for T cell responses for an additional four months after signing the informed consent. Peripheral blood samples were collected from the placebo group and from the individuals who were vaccinated with the inactivated SARS-CoV-2 vaccine (CoronaVac, Sinovac Life Sciences Beijing, China) on the days 7 and 120 following the second dose. CoronaVac consists of β-propiolactone-inactivated whole-virion of SARS-CoV-2 CZ02 strain (3 μg/0.5 mL/dose), which is produced in African green monkey kidney (Vero) cells, and aluminum hydroxide (0.45 mg/mL) as adjuvant^23^. The placebo contained all ingredients except the inactivated virus. The volunteers received two intramuscular administrations 14 days apart. Information on the exclusion and eligibility criteria, the study cohort design and randomization were published in a previous study^22^. The participants were 18–59 age-old with no history of COVID-19 (Table 1). A portion of the volunteers (n = 31) could be followed on day 7 and day 120 (data presented as dependent variables); however, other participants (n = 26 on day 7; n = 65 on day 120) were enrolled to the study with a cross-sectional approach. The study was commenced following the approvals by the local ethical committee (approval no: 2020/10-26 and 2020/17-31) and the Republic of Turkey Ministry of Health. The first registration date is Nov 11, 2020, for the phase 3 clinical trial, and this study covers a follow-up period between on Jan 15, 2021, and Jul 01, 2021. All experiments were performed at Hacettepe University, Ankara, Turkey in accordance with the relevant guidelines and regulation. A signed informed consent was obtained from the volunteers.

**Table 1:** Data of the volunteers enrolled in the study.

	CoronaVac (day 7)	CoronaVac (day 120)	Placebo
Number (n)	57	96	38
Age median (range)	45 (18–59)	43 (19–59)	43 (18–57)
Gender female/male	31/26	57/39	17/21

### Generation of monocyte-derived DC (mDCs) and co-culture experiments

As a T cell functional analysis method established by our group, the S1-loaded mDCs were co-cultured with the autologous memory T cell subsets freshly purified from the same individual. In this co-culture method, the specificity of T cell responses against S1 protein was previously confirmed by comparing the results obtained with mDCs loaded with an unrelated protein antigen (HIV Gag) and with mDCs which were not loaded with a specific protein^24^. Briefly, peripheral blood mononuclear cells (PBMCs) were separated by density gradient centrifugation (Ficoll 1.077 g/mL, Sigma). CD14^+^ monocytes were isolated by MACS (Miltenyi, Gladbach, Germany) and monocyte-derived dendritic cells (mDCs) were generated according to a published protocol^25^. Maturation of monocyte-derived DCs was induced by LPS (1 µg/mL; Sigma) simultaneously with the recombinant S1 spike glycoprotein (10 µg/mL; Abcam) antigen loading (S1-mDC). The S1 antigen used is a 235 amino acid-long commercially available immunodominant peptide which covers a region highly shared by the SARS-CoV-2 variants. The mDCs generated in the absence of the S1 protein were used as “no specific antigen-loading” controls. After 7 days of incubation, the CD11b^hi^CD14^lo^CD1a^+^CD83^+^ mDC population was confirmed by immunophenotyping (Suppl. Fig. S1).

Prior to the establishment of the co-cultures, the memory T cell subsets were purified from the peripheral blood samples freshly obtained from the volunteers enrolled to the study. CD19^-^CD56^-^ lymphocytes were gated and the terminally-differentiated effector T (CD45RA^+^CD45RO^-^CCR7^lo^, T_EMRA_), the central memory T (CD45RA^-^CD45RO^+^CCR7^hi^, T_CM_), and the effector memory T (CD45RA^-^CD45RO^+^CCR7^lo^, T_EM_) cells were isolated (with a purity ≥ 96%) by using a fluorescence-activated cell sorter (FACSAria II, Becton Dickinson) (Suppl. Fig. S2). The T cells (10^5^) were labelled with carboxyfluorescein succinimidyl ester (CFSE, 5 µM; BioLegend) and incubated with the mDCs (5 × 10^4^) and recombinant IL-2 (5 ng/mL; BioLegend) for 96 h. The co-cultures established in the presence of anti-CD3 monoclonal antibody (HIT3a, 25 ng/mL; BioLegend) served as a positive control. All cell cultures were performed in RPMI 1640 medium supplemented with 10% fetal bovine serum (FBS) and 1% penicillin–streptomycin (Biological Industries). The cultures were kept at 37 °C in a humidified incubator with 5% CO_2_.

### Flow cytometry

The monoclonal antibodies anti-human-CD11b (ICRF44), -CD14 (M5E2), -CD19 (HIB19), -CD56 (B159), -CD45RA (HI100), -CD45RO (UCHL1), -CCR7 (2-L1-A), -CD4 (RPA-T4), -CD8 (HIT8A), -CD25 (M-A251), -CD38 (HB7), -4-1BB (4B4-1), -PD-1 (EH12.1), -CD107a (H4A3) (Beckton Dickinson); CD1a (REA736), -CD83 (REA714) (Miltenyi) were used for immunophenotyping of T cells and/or DCs. T cell proliferation was assessed according to the CFSE fluorescence dilution. The change in proliferation was calculated by normalizing the percentage of proliferated T cells from the co-cultures with the S1-mDC to that of obtained from the non-specific antigen-loaded mDC co-cultures. Median fluorescence intensity (MFI) and percentage values were obtained from the events gated on CD4^+^ and CD4^−^ T cells following the co-culturing with the mDCs. The gating strategy used is shown in Suppl Fig. S3. MFI values from specific subpopulations were normalized to those of from total events counted and represented as a heat-map. The samples were run and analyzed on a FACSCanto II (Becton Dickinson) flow cytometer.

### Cytometric bead assay

The supernatants were collected from the co-cultures at 96 h. IFN-γ levels were measured by a flow cytometry-based bead assay (LEGENDPlex™ Human IFN-γ, BioLegend). MFI values of the samples and the standards were used for calculating the concentration of IFN-γ (LEGENDplex™ Data Analysis Software, BioLegend) as instructed by the manufacturer.

### Statistical analysis

The data are presented as median with standard error of the mean (SEM). Student’s paired or unpaired t-test or One-Way ANOVA were used for the statistical analysis. The central tendencies of the groups were determined by the Mann–Whitney U test. When P value was < 0.05, it was considered as statistically significant.

## Results

### Exposure to SARS-CoV-2 glycoprotein S1 evokes CD4 and CD8 memory T cell proliferation in vaccinated individuals

**Figure 1:**
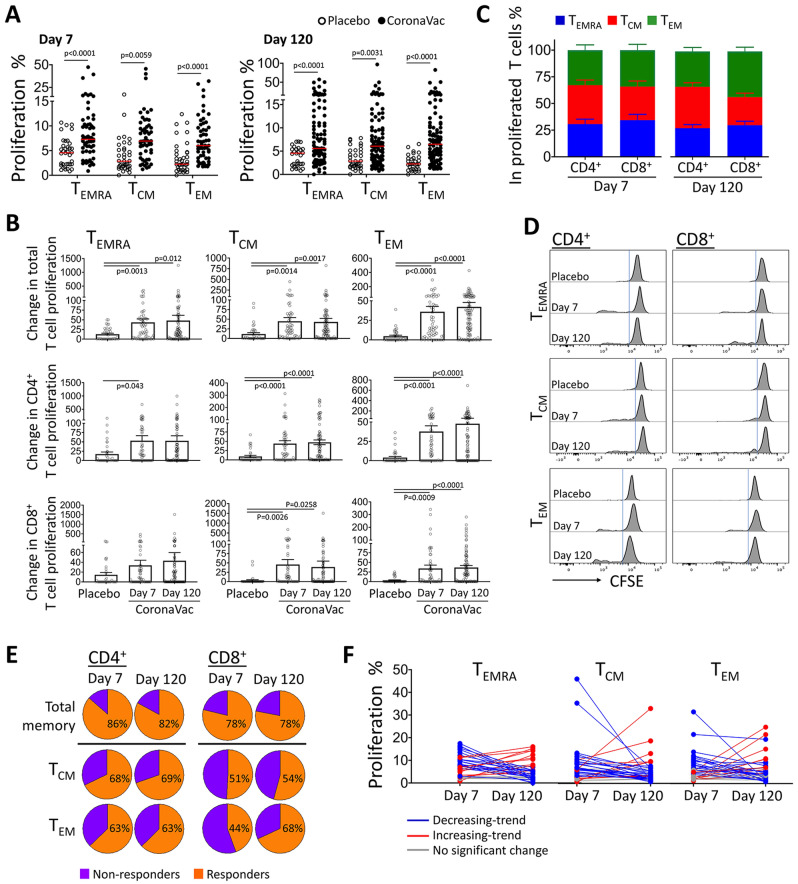
Proliferation responses in memory T cells from the individuals after the aluminum-adjuvanted inactivated whole-virion SARS-CoV-2 vaccination demonstrated a considerable response against S1 antigen. Autologous monocyte-derived dendritic cells were loaded with SARS-CoV-2 Spike Glycoprotein-S1 and co-cultured with terminally-differentiated effector T (T_EMRA_), central memory T (T_CM_), and effector memory T (T_EM_) cells purified from the individuals after the second dose of CoronaVac (n = 48 on day 7 and n = 81 on day 120) or placebo administration (n = 34) on day 7 and day 120. Data obtained from the experiments without any technical complications are plotted; therefore, the number of volunteers for each group do not match to those of the total number of participants enrolled to the study. (**A**) Percentage of proliferation in memory T cell subsets is shown for each case. (**B**) Change in CD4^+^ and CD8^+^ memory T cell proliferation was calculated in comparison to the T cells co-cultured with non-specific antigen-loaded mDCs (without S1 antigen loading). (**C**) Contribution of each memory T cell subset to the overall proliferation response in CD4^+^ and CD8^+^ cells. (**D**) Flow cytometry histograms displaying representative proliferation responses from T cell subtypes are shown. (**E**) The percentage of participants whose T cells were responded to S1-loaded mDCs and proliferated more than the median value of the placebo group is demonstrated as “responders”. (**F**) The proliferation of T_EMRA_, T_CM_, and T_EM_ obtained on day 7 and day 120 is given on individual basis (n = 32). (Significance was determined by Mann–Whitney U test for (**A**) and One-Way ANOVA for (B), *p < 0.05, **p < 0.01). Each dot represents a single participant.

In order to determine the functional capacities of memory T cell subsets, T_EMRA,_ T_CM_ and T_EM_ obtained from the individuals vaccinated with the aluminum-adjuvanted inactivated whole-virion SARS-CoV-2, CoronaVac, we employed a laborious co-culture strategy previously validated for screening the specific anti-S1 T cell responses^26^. Briefly, CD4^+^ and CD8^+^ T_EMRA,_ T_CM_ and T_EM_ cell subsets were collected on 7th and 120th days following the second dose of the vaccine and were exposed to S1 antigen-loaded autologous monocyte-derived DCs. Proliferation of all three memory subsets was significantly higher in the CoronaVac group when compared to that of in the placebo group. There was no significant difference when the proliferation responses of the T cells obtained on day 7 and day 120 were compared (Fig. 1A,B). The change in proliferation rate was also comparable between CD4^+^ and CD8^+^ T_CM_ and T_EM_ cells from the vaccinated individuals. In contrast to CD4^+^ T_EMRA_ cells, the change in CD8^+^ T_EMRA_ proliferation did not reach to the level of statistical significance (Fig. 1B). On the other hand, each memory T cell subset contributed to the total T cell proliferation (Fig. 1C). Only, CD8^+^ T_EM_ cells tended to have a higher proliferating fraction than the other two subsets on day 120 (Fig. 1C,D).

Proliferation response by memory CD4^+^ helper T cells or CD8^+^ cytotoxic T cells was detected both on day 7 and 120 in the majority (> 78%) of the individuals vaccinated with CoronaVac. More than 63% of the individuals was identified with a CD4^+^ T_CM_ and T_EM_ response. Even though percentages of the individuals whose CD8^+^ T_CM_ and T_EM_ cells responded to S1-loaded DCs on day 7 were 51% and 44%, respectively, the number of responders was increased on day 120 (Fig. 1E). Expectedly, the proliferation capacity of T_EMRA,_ T_CM_ and T_EM_ cells were modulated between day 7 and day 120. At individual level, in the majority of the responders, a decreasing trend was observed when the proliferation of memory subsets obtained on day 7 was compared to that of on day 120. Alternatively, the memory T cells from several vaccinated individuals displayed higher proliferation response on day 120 (Fig. 1F).

Collectively, majority of the individuals who undergone aluminum-adjuvanted inactivated whole-virion SARS-CoV-2 vaccination was identified with CD4^+^ and CD8^+^ memory T cell responses. Compared to the placebo group, T_EMRA,_ T_CM_ and T_EM_ cells compatibly proliferated in response to S1-loaded mDCs. Albeit being heterogeneous, the memory T cell proliferation rate tended to decrease in the vaccinated individuals.

### Modulation of IFN-γ secretion and activation capacity of memory T cells after vaccination

IFN-γ secretion capacity of antigen-specific T cells can serve as a measure for a successful anti-viral immunity^27^. Additionally, expression of certain surface molecules can indicate T cell activation and effector functions. Therefore, IFN-γ secretion and upregulation of CD25, CD38, 4-1BB, PD-1, and CD107a molecules were assessed on the memory T cell subsets that were co-cultured with the S1-loaded mDCs.

**Figure 2:**
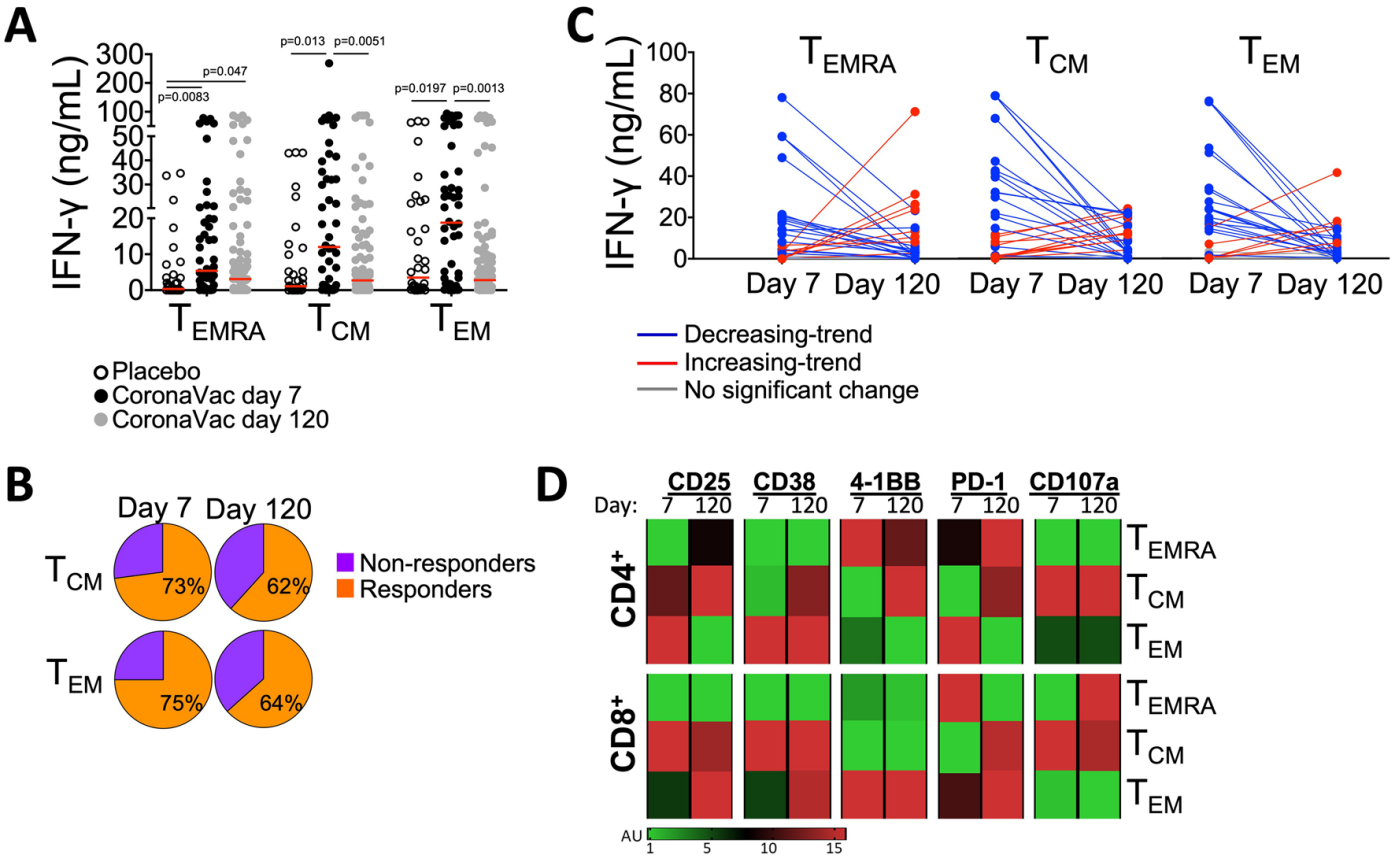
Modulation of IFN-γ secretion and activation markers on memory T cell subtypes indicated the efficacy of whole virion vaccination on cellular immunity. T_EMRA_, T_CM_, and T_EM_ obtained from the placebo group and the CoronaVac group on day 7 and day 120 were co-cultured with the S1-loaded mDCs. IFN-γ secreted into the supernatants and the expression of CD25, CD38, 4-1BB, PD-1, and CD107a markers were assessed by flow cytometry-based methods after 96 h of incubation. (**A**) The amount of IFN-γ secreted by memory T cells is shown. (**B**) The percentage of participants whose T cells were responded to the S1-loaded mDCs and secreted more IFN-γ than the median value of the placebo group is demonstrated as “responders”. (**C**) The IFN-γ secretion capacity of T_EMRA_, T_CM_, and T_EM_ obtained on day 7 and day 120 is given on individual basis (n = 31). (**D**) The change in the expression levels of activation markers for the CD4^+^ and CD8^+^ T memory cells on day 7 (n = 56) and day 120 (n = 46) is presented in a heat map and displayed as arbitrary units (A.U) which were calculated in comparison to those obtained from the co-cultures with control mDCs. Data obtained from the experiments without any technical complications are plotted; therefore, the number of volunteers for each group do not match to those of the total number of participants enrolled to the study. (Significance was determined by Mann–Whitney U test for (**A**), *p < 0.05, **p < 0.01). Each dot represents a single participant.

The amount of IFN-γ secreted by the memory T cells obtained on day 7 after the vaccination was significantly higher than the placebo group. The highest levels of IFN-γ were detected in the co-cultures with T_EM_ cells followed by T_CM_ and T_EMRA_ (Fig. 2A). On day 120, the T_EMRA_ cells from the vaccinated individuals continued to secrete significantly more IFN-γ than the cells obtained from the placebo group. Of note, both on day 7 and day 120, a group of vaccinated individuals harbored memory T cell subsets that recalled the S1 antigen and produced considerable amounts of IFN-γ (Fig. 2A). When the median IFN-γ concentration of the placebo group was considered as a threshold, more than 73% (on day 7) and 62% (on day 120) of the vaccinated individuals contained T_CM_ and/or T_EM_ cells that responded to S1-loaded mDCs by secreting IFN-γ (Fig. 2B). Nevertheless, except for a few cases, there was a decreasing trend in the amount of IFN-γ produced by the memory T cells when the data acquired for day 7 and day 120 were compared (Fig. 2C).

The expression of CD25, CD38, 4-1BB, PD-1, and CD107a indicated the modulation of S1-specific CD4^+^ and CD8^+^ memory T cell responses on day 7 and day 120. In general, these molecules were mostly altered on CD4^+^ T_EM_, CD4^+^ T_CM,_ and CD8^+^ T_EM_ cells upon stimulation with the S1-loaded mDCs. In contrast to the CD4^+^ T_EM_ cells, which displayed high levels of the immune modulatory molecules on day 7, CD4^+^ T_CM_ and CD8^+^ T_EM_ cells tended to increase the expression of these surface markers on day 120 (Fig. 2D). Notably on day 120, CD25, PD-1, 4-1BB, and CD38 were upregulated on CD4^+^ T_CM_; CD25, PD-1, and CD38 were upregulated on CD8^+^ T_EM_ cells, and CD25 and PD-1 were upregulated on CD4^+^ T_EMRA_ cells (Fig. 2D). CD4^+^ T_EM_ cells displayed high CD25, PD-1, and 4-1BB expression on day 7. Except for CD8^+^ T_EMRA_ cells, PD-1 expression was generally increased on all memory T cell subtypes on day 120 (Fig. 2D). CD107a, which is a marker for degranulation and cytotoxicity, was more abundantly expressed only on CD8^+^ T_EMRA_ cells (Fig. 2D, Suppl. Fig. S4).

Collectively, in response to the S1-loaded mDC stimulation, the memory T cells collected on day 120 after the second CoronaVac dose secreted lower amounts of IFN-γ, and frequently upregulated the inhibitory receptor PD-1.

## Discussion

The inactivated whole-virion SARS-CoV-2 vaccine, CoronaVac, has a good safety profile and can induce considerable humoral immunity against SARS-CoV-2^22^. According to the interim results from a recent phase III clinical trial in Turkey, the vaccination highly prevented symptomatic disease (83.5% relative to placebo) and COVID-19-related hospitalization at least for 14 days after the second dose^22^. A great majority of the volunteers developed anti-spike protein receptor-binding domain (S-RBD) antibodies with virus neutralization capacities^28^. Assessment of functional capacities of memory T cells is pivotal for estimating the efficacy of an immunization^29,30^. Here, we report the potential of this aluminum-adjuvanted inactivated whole-virion vaccine for establishing an intermediate-term T cell immunity and demonstrates capacity of the vaccine-induced memory T cells to inaugurate functional responses against the SARS-CoV-2 spike glycoprotein S1. In contrast to the previous work on different vaccines for COVID-19, which applied cross-sectional analyses such as intracellular cytokine staining (ICS), ELISPOT and immunophenotyping, the methodology used in our study not only determines the presence of distinct subsets of memory T cells but also enables a real-time quantification for the functional consequences of S1-specific recall stimulation^26^.

The mRNA-based vaccines such as BNT162b2 (Pfizer-BioNTech) and mRNA-1273 (Moderna) or the viral vector-based vaccines such as ChAdOx1 nCoV-19 (AZD1222, Oxford/AstraZeneca), Gam-COVID-Vac (Sputnik V, Gamaleya Inst.) and Ad26.COV2.S (Johnson & Johnson) take the advantage of providing the SARS-CoV-2 S-RBD antigen directly through intracellular antigen processing and cross-presentation pathways which enable the CD4^+^ and CD8^+^ T cell reactions^31–34^. All these vaccination strategies put the magnitude of cellular immunity and memory T cell responses forward as a measure of efficacy and longevity of protection against COVID-19^35^. Nevertheless, protective immune responses can also be achieved by inactivated whole-virion vaccines for influenza and SARS-CoV-2^36–38^. Correspondingly, our results demonstrated that T_EMRA_, T_CM_ and T_EM_ memory T cells reactive for the S1 antigen of the SARS-CoV-2 develop in the individuals vaccinated with CoronaVac as early as 7 days after the second dose. In preclinical models, the antibody responses induced by CoronaVac and several other inactivated whole-virion vaccine candidates effectively neutralized SARS-CoV-2 strains and enabled protection against SARS-CoV-2 challenge^26,39^.

The COVID-19 tend to show transition to endemicity, lose virulence and exhibit less severe infections^40^. Sustaining high levels of population immunity would lower the risk of a new pandemic transmitted with the SARS-CoV-2 variants of concern (VOC). Through the endemic perspective, the immunity against SARS-CoV-2, which will eventually wane, needs to be precisely monitored^41^. Therefore, the memory responses to the shared antigens amongst VOC become more critical for maintaining the immunity against COVID-19. Even though S-RBD has been regarded as a critical antigen for protective immunity through neutralizing antibodies, the IgG responses in COVID-19 patients are frequently specific to non-RBD epitopes of the spike protein and to other structural components such as matrix proteins, envelope proteins, and nucleoprotein^42,43^. In the individuals immunized with a whole-virion vaccine, T cell immunity against a plethora of other antigens such as nucleocapsid (N) and envelope (E) molecules might be generated^44^. As a limitation of our study, we focused on the immunity elicited to S1 while it is still important to determine if T cells react to other SARS-CoV-2 antigens.

The presence of a long-term T cell memory in COVID-19 patients has been validated^45–47^. Moreover, in a previous work using a similar methodology, we have demonstrated the S1-specific T_CM_ and T_EM_ responses being prominent in the individuals with COVID-19 history^24^. Even though the immune responses elucidated with the inactivated whole-virion vaccines cannot be restricted to the spike protein, here, the immunodominant region of S1 protein was used as a preferred SARS-CoV-2 antigen and loaded to the autologous mDCs to test the S1-specific T cell response^24^. Accordingly, the functional potency of T cell memory against the S1 protein has been considered critical for the longevity of B cell responses and the affinity maturation of antibodies reactive for the spike region^48^. In our study, the CD4^+^ and CD8^+^ T_EMRA_, T_CM_, and T_EM_ cells obtained from the volunteers vaccinated with CoronaVac not only proliferated and secreted IFN-γ in response to S1-loaded mDCs but also upregulated the surface molecules associated with activation and immune regulation.

Basically, the increased levels of these molecules on the S1-stimulated memory T cells can be used as an indicator of stimulation and as a correlate of immunity inaugurated with CoronaVac. The memory T cell pool serves as a dynamic repository of antigen-experienced T cells. T_EM_ cells are distinguished with rapid effector functions. T_CM_ cells express homing receptors for migration to secondary lymphoid organs where they can easily interact with antigen presenting cells and B lymphocytes^49^. The increased upregulation of T cell activation markers on the T_CM_ subset especially at day 120 may indicate the longevity of T cell mediated memory and these cells’ capacity to contribute to the immune reactions in the lymph nodes. On the other hand, PD-1 positivity in the memory T cells may indicate the modulation of immune responses especially at late periods. As an inhibitory receptor, PD-1 dampens the effector responses of T cells and restricts exacerbation of the inflammatory responses in order to avoid collateral damage^50^. The upregulation of immune regulatory molecules might have contributed to the decline of proliferation and IFN-γ secretion capacities in memory T cell subsets when re-stimulated with S1-loaded mDCs on day 120.

Particulate adjuvants such as aluminum salts create a local inflammatory environment and induce the recruitment of innate immune cells which mediate the uptake and presentation of the antigen^51^. Aluminum adjuvants support IgG production and tend to increase type 2 helper T (Th2) responses^52^. On the other hand, the anti-viral immunity not only relies on neutralizing antibodies, but also potent Th1 and CTL responses are required^53,54^. Moreover, the follicular helper T (Tfh) cells have improved ability to interact with B lymphocytes and support germinal center reactions. Many reports have linked the success of vaccination with Tfh functions^55,56^. As a drawback, our study did not focus on the memory responses-related to specific Th subtypes. The neutralizing antibody titers determined in the volunteers who were enrolled in this study were not directly correlated with the functional memory T cell responses (data not shown). Nevertheless, the longevity of protection from viral infections is directly associated with generation of memory B cells together with CD4^+^ and CD8^+^ memory T cells^57^. Albeit being a frequent component of many human vaccines, alum has been acknowledged with low capacities to induce T cell memory^52^. Nevertheless, the nature of antigen has a significant impact on T cell-mediated immunity^58^. Inactivated whole-virion vaccines provide a complex antigenic structure which enhances antigen presentation and T cell responses^59^. Even though there is no consensus in the literature, alum can also positively influence DC maturation and/or increase the costimulatory signals for T cell-mediated immunity^53^. BBV152 (Bharat Biotech), which contains an inactivated SARS-CoV-2 virion together with alum and toll-like receptor (TLR)7/8 agonist IMDG, induced type 1 helper T (Th1) responses, nevertheless seroconversion rates were moderate in a phase 1 trial^60^. In accordance with our results, another aluminum-adjuvanted inactivated whole-virion SARS-CoV-2 vaccine, Sinopharm, was also reported to induce CD107a and IFN-γ expression in CD4^+^ and CD8^+^ T cells^61^. Therefore, combination of an inactivated whole-virion particles with the aluminum adjuvants possesses capacities to induce T cell responses and support the generation of memory T cell subsets.

In conclusion, the aluminum-adjuvanted inactivated whole-virion SARS-CoV-2 vaccine, CoronaVac, induced functional S1-specific memory T cell responses. Either after a short-term or an intermediate-term following the second dose of the vaccine, majority of the individuals harbored CD4^+^ and CD8^+^ memory T cell subsets that proliferated and secreted IFN-γ in response to the S1 antigen. Even though an immune modulation was noted together with a decline in the responsiveness after 4 months, the overall memory T cell reactions demonstrated the efficiency of aluminum-adjuvanted inactivated whole-virion vaccines to induce immunological memory.

## Supplementary Information

Supplementary Figures.

## Data Availability

For original data, please contact gunese@hacettepe.edu.tr.
