# Supplementary material for: Human memory T cell dynamics after aluminum-adjuvanted inactivated whole-virion SARS-CoV-2 vaccination
Source: Sci Rep. 2023 Mar 21;13:4610. doi: 10.1038/s41598-023-31347-8 (PMC10028771; doi:10.1038/s41598-023-31347-8)
Supplement: Supplementary file 1 — Supplementary Figures. [file 41598_2023_31347_MOESM1_ESM.docx]

**Supplementary Figures**


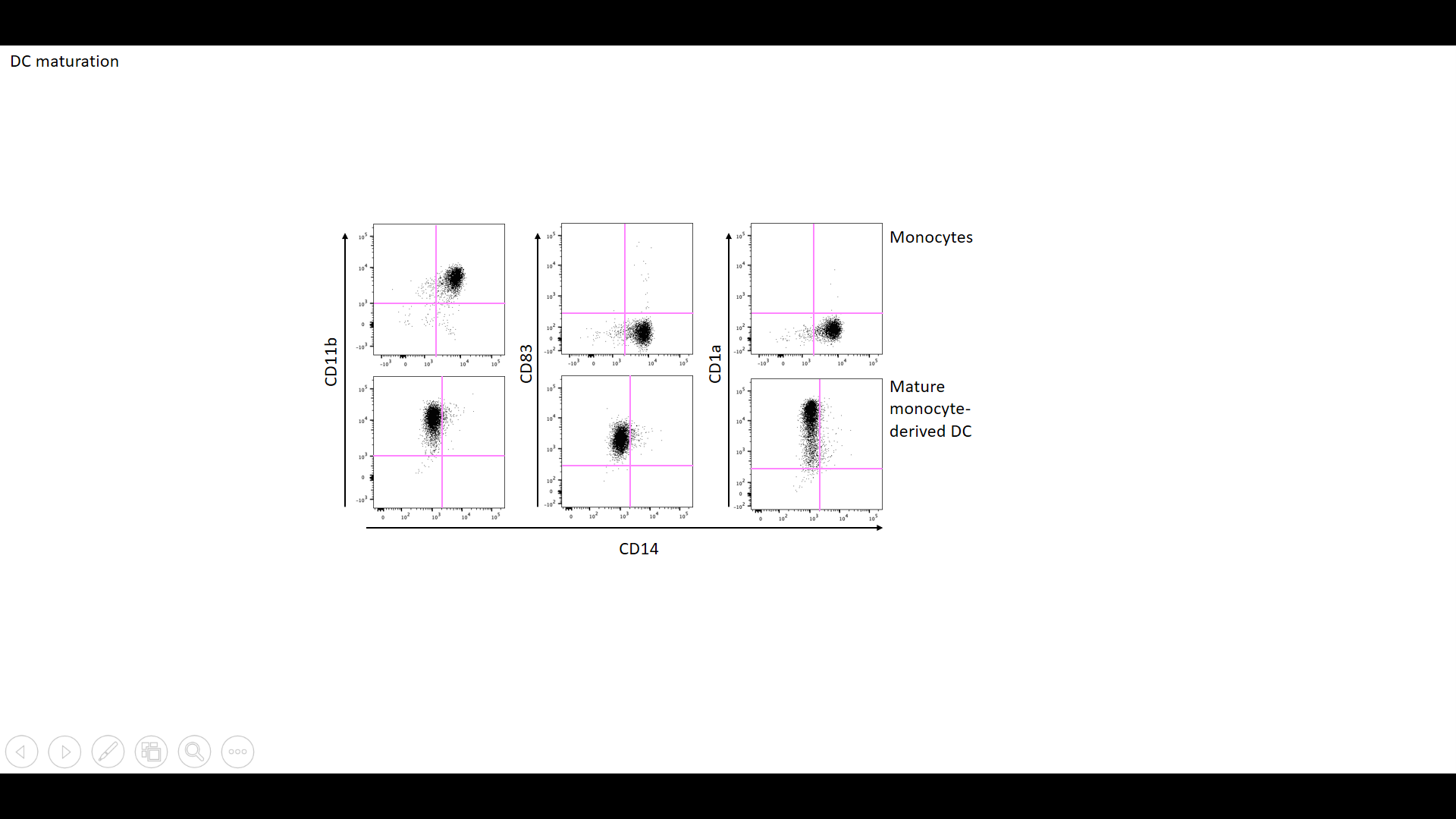


**Supplementary Figure 1.** Confirmation of mature monocyte-derived dendritic cells (mDCs) loaded with SARS-CoV-2 S1 protein was performed flow cytometric immunophenotyping. Results from mDCs generated from the peripheral blood monocytes obtained from a volunteer enrolled to the study are represented.


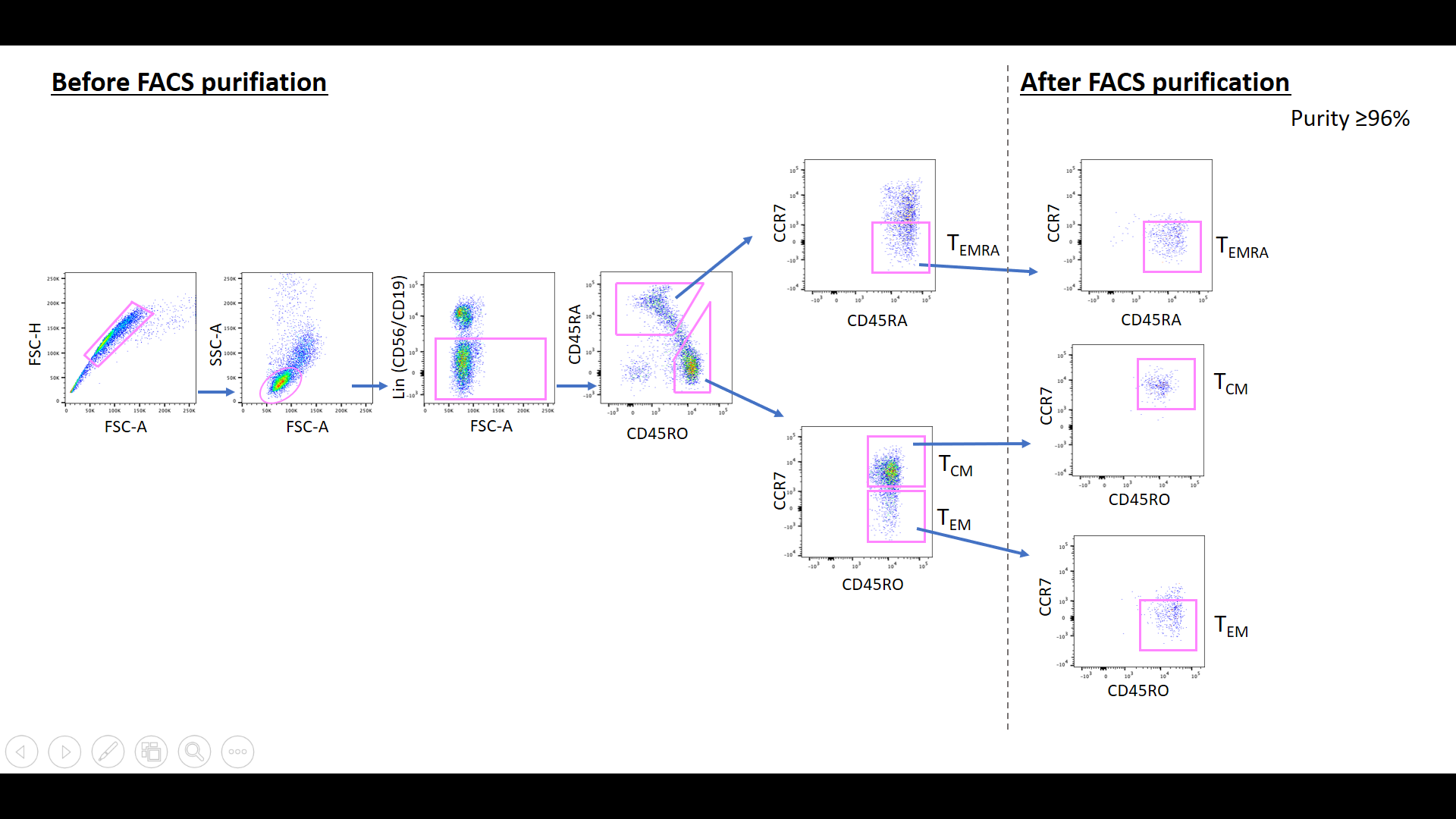


**Supplementary Figure 2.** Purification strategy for memory T cell subsets by FACS. Peripheral blood mononuclear cells (PBMCs) isolated from the volunteers were gated as CD56/CD16-negative cells, distributed according to CD45RA and CD45RO isoforms, and sorted according to CCR7 expression. The freshly purified T cells were used in the co-cultures with autologous mDCs for functional analyses.


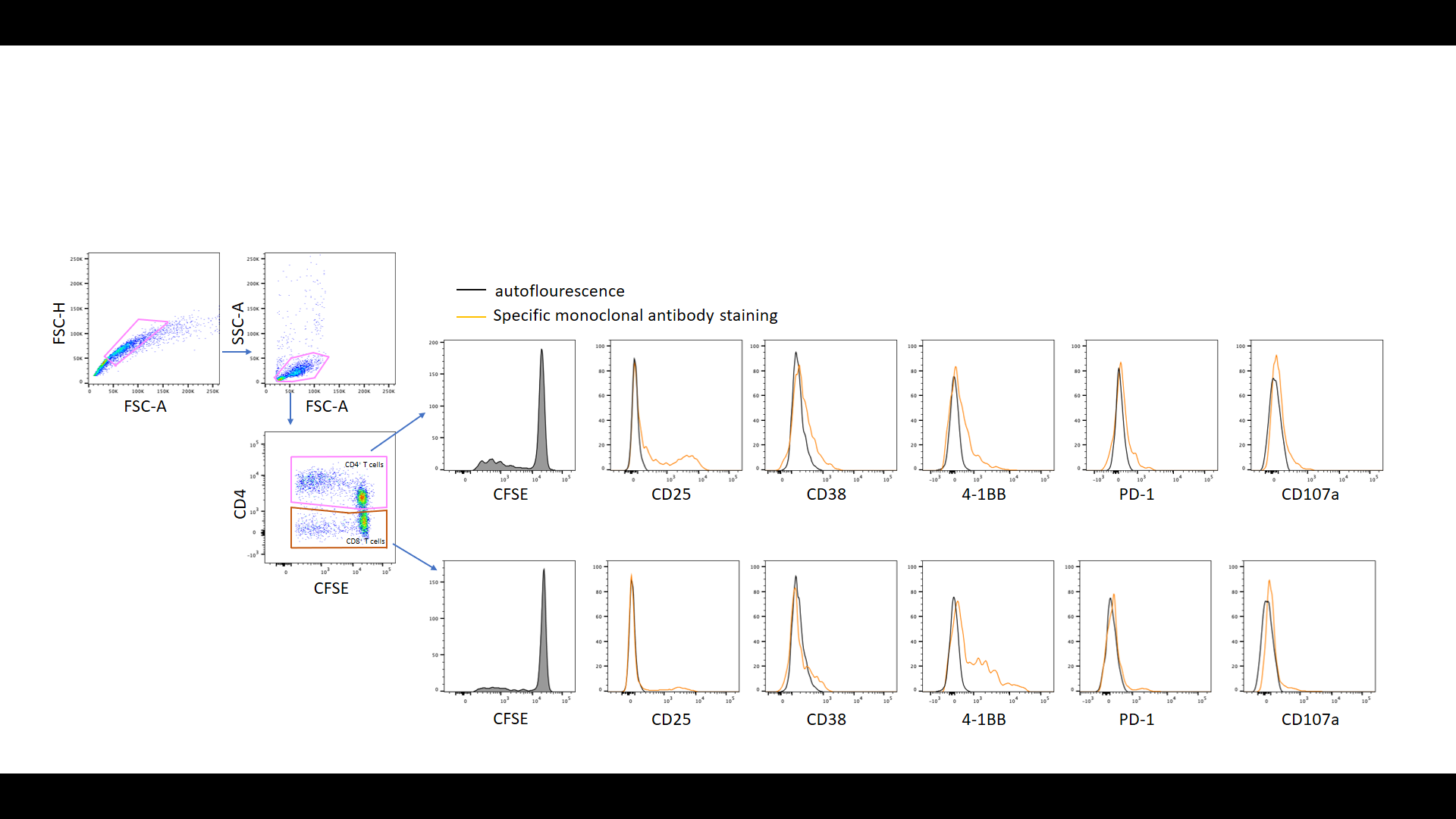


**Supplementary Figure 3.** Analysis strategy following the co-culturing of the T cell memory subsets and autologous mDCs from the vaccinated individuals. CFSE-labelled T cells were gated CD4^-^ (CD8^+^) and CD4^+^ subtypes. The percentage of cells with diluted CFSE fluorescence and the expression of activation markers were assessed by flow cytometry. Please note that the immunophenotyping was performed on the total population of T cells gated in the co-cultures.


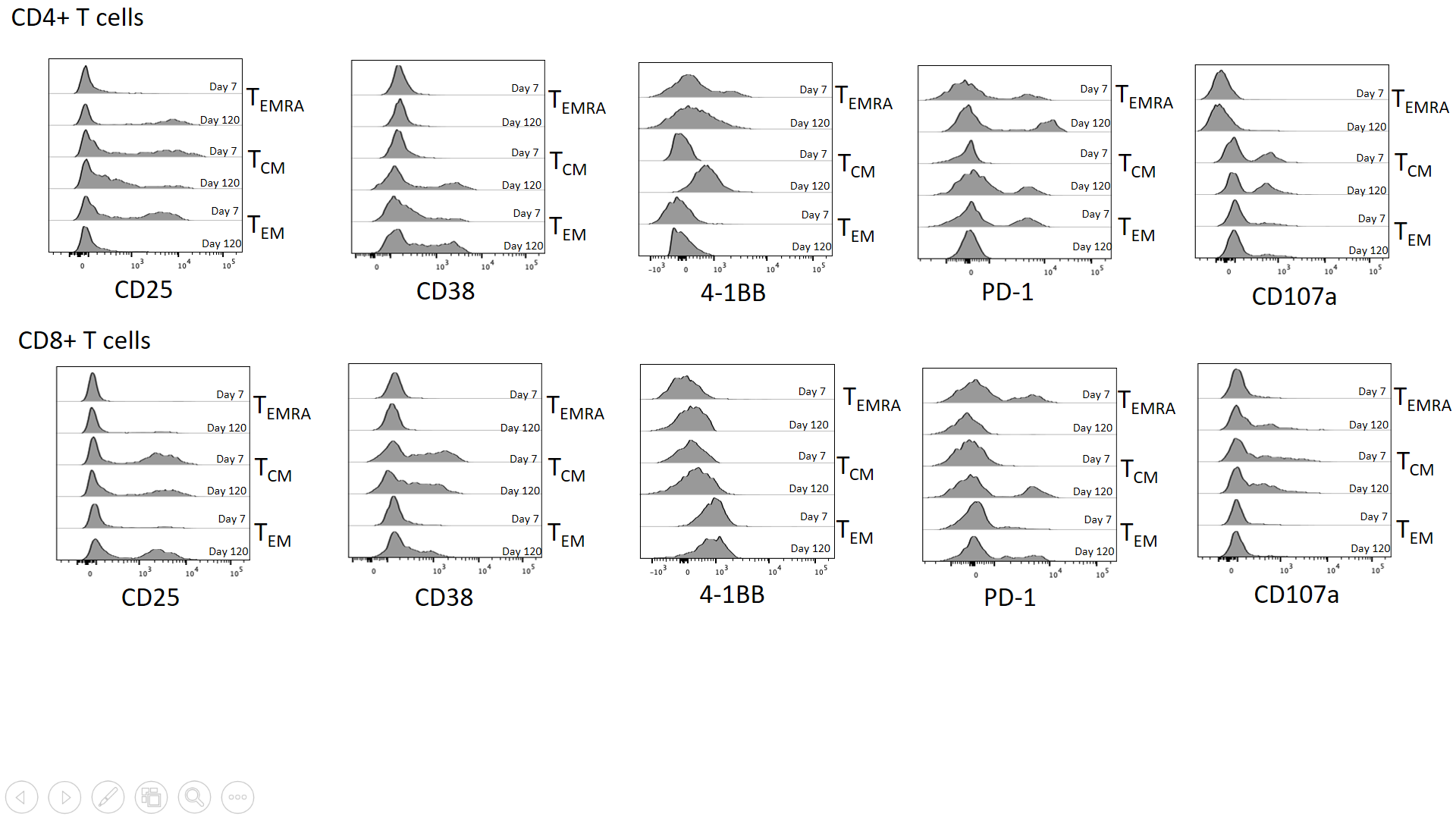


**Supplementary Figure 4.** Representative flow cytometry histograms showing the expression of activation markers on CD4^+^ or CD8^+^ T_EMRA_, T_CM_ and T_EM_ cells collected from the vaccinated volunteers on day 7 and day 120 and co-cultured with S1-loaded mDCs.
